# Transforming Brain Organoids Into Functional Platforms: Convergence of Engineering, Imaging, AI, and Ethics

**DOI:** 10.1002/advs.202523506

**Published:** 2026-09-29

**Authors:** Aishwarya Pantula, Kaustubh Joshi, Swarangi Samant, Greta Babakhanova, Annie Kathuria

**Affiliations:** ^1^ Department of Biomedical Engineering Whiting School of Engineering Johns Hopkins University Baltimore Maryland USA; ^2^ Department of Biotechnology Advanced Academic Programs Krieger School of Arts and Sciences Johns Hopkins University Baltimore Maryland USA; ^3^ Biosystems and Biomaterials Division National Institute of Standards and Technology Gaithersburg Maryland USA; ^4^ Department of Neurosurgery School of Medicine Johns Hopkins Medicine Baltimore Maryland USA

**Keywords:** AI and ethics, brain organoids, microphysiological systems, multiomics, organoid‐on‐a‐chip

## Abstract

Brain organoids are self‐organizing 3D aggregates derived from pluripotent stem cells and thus mirror many aspects of human brain development, including gene expression programs, spatial organization, and neuron subtype specification. As a result, these organoids have been used to emulate neurodevelopmental, neuropsychiatric, and neurodegenerative diseases. Yet, their maturation remains fundamentally constrained by diffusion‐limited necrosis, incomplete vascularization, limited visualization, and the absence of large‐scale circuit integration, which collectively prevent progression beyond the fetal stage. These intrinsic limitations have catalyzed a convergence across microfluidics, biofabrication, imaging, and ethical frameworks, giving rise to a multidisciplinary movement aimed at enhancing organoid viability, reproducibility, and physiological relevance. This perspective examines how these technologies are transforming brain organoids from developmental models into experimentally robust, functional platforms.

## Graphical abstract

Brain organoids are emerging as increasingly functional human‐relevant platforms, yet limitations in vascularization, maturation, measurement, and reproducibility still constrain their translational value. This Perspective examines how microfluidics, biofabrication, advanced imaging, electrophysiology, multimodal AI, standardization, and ethical governance converge to address these bottlenecks and define the next steps toward robust, interpretable, and experimentally useful brain organoid systems.

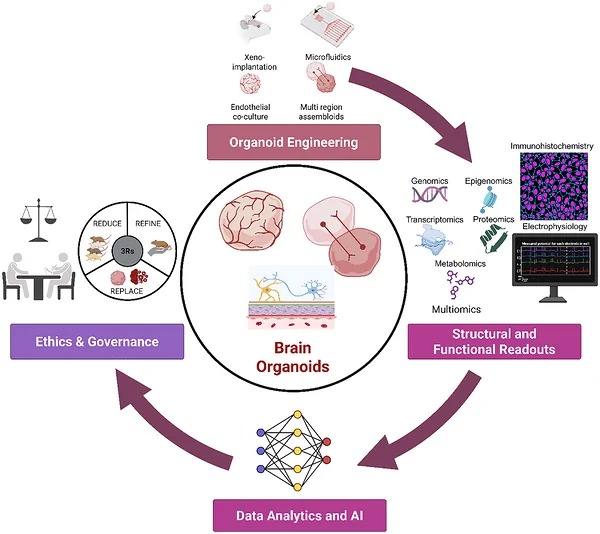

## 1 Introduction

Deciphering human brain development and the origins of neurological disease remains a central challenge in biology [1]. The intricate architecture of neural circuits and the limited accessibility of human brain tissue have long impeded our understanding of underlying mechanisms. Decades of research in developmental neuroscience have revealed a precise sequence of events: neural progenitors proliferate under morphogen control [2, 3], neurons migrate along glial scaffolds [4], differentiate into specific subtypes, and form synaptic connections guided by extracellular cues. These circuits undergo refinement through activity‐dependent pruning and microglial clearance [5], while oligodendrocytes [6] initiate myelination to improve conduction. Synaptic plasticity continues to remodel these networks [7, 8]. Disruptions at any stage may lead to neurodevelopmental disorders or increase the risk of late‐onset neurological diseases.

Human brain tissue obtained through surgery or post‐mortem collection offers a valuable biological context but presents major drawbacks. Differences in donor genetics, environmental exposures, and the variability of preservation techniques often make interpretation difficult. To circumvent these challenges, animal models have long served as the cornerstone of experimental neuroscience, illuminating conserved principles of brain structure and function across evolution. Yet despite their utility, they often fail to capture hallmark features of human neurological disease, such as gradual neuronal loss, complex glial‐immune interactions, and human‐specific signaling cascades, limiting their translational relevance and predictive power [9, 10]. Recent regulatory movements to reduce additional animal testing reflect both ethical considerations and a growing recognition of these scientific limitations. Together, these factors underscore the need for more human‐relevant platforms [11, 12]. Among these new alternatives, induced pluripotent stem cells (iPSCs) in particular have gained prominence for their potential to offer more physiologically relevant insights into disease mechanisms and drug responses.

The discovery by Takahashi and Yamanaka in 2007 [13] that somatic cells can be reprogrammed into iPSCs revolutionized human in vitro modeling. iPSCs not only bypass the ethical constraints of embryonic cells [13] but also retain the donor's genetic background, enabling patient‐specific studies of development, disease mechanisms, and therapeutic responses. However, most early iPSC‐based models relied on 2D monolayer cultures, which, while useful for basic differentiation and molecular assays, fail to replicate the microenvironmental and multicellular complexity of native tissues [14]. The absence of physicochemical gradients, cell‐cell and cell‐matrix interactions, and 3D architecture limits their ability to mimic lineage specification and morphogenesis.

Consequently, 2D models misrepresent differentiation trajectories and signaling dynamics, prompting the rise of organoids and 3D microphysiological systems (MPS) as microscale cell‐culture platforms to model the functional features of a specific tissue or organ of human or animal origin by exposing cells to a microenvironment that closely mimics the physiological aspects critical to their function or pathophysiological condition [15, 16].

Despite rapid advances in organoid generation, the central challenge is no longer simply producing increasingly complex neural tissues but overcoming interconnected technological bottlenecks that limit maturation and experimental utility to meet regulatory demands. Diffusion‐limited transport and incomplete vascularization constrain tissue size and long‐term viability; incomplete regional organization and circuit integration restrict modeling of coordinated neural function; limited access to deep and longitudinal functional readouts complicates assessment of maturation; and inter‐organoid and inter‐batch variability limits reproducibility.

This perspective examines how convergent advances in microfluidics, biofabrication, functional imaging, and computational analysis are addressing the fundamental constraints that have limited organoid maturation and physiological relevance. By integrating these multidisciplinary approaches with evolving ethical frameworks, the field is positioned to transform brain organoids from developmental models into robust experimental platforms that can model disease mechanisms, test therapeutics, and advance our understanding of human neurobiology. (Figure 1).

**FIGURE 1:**
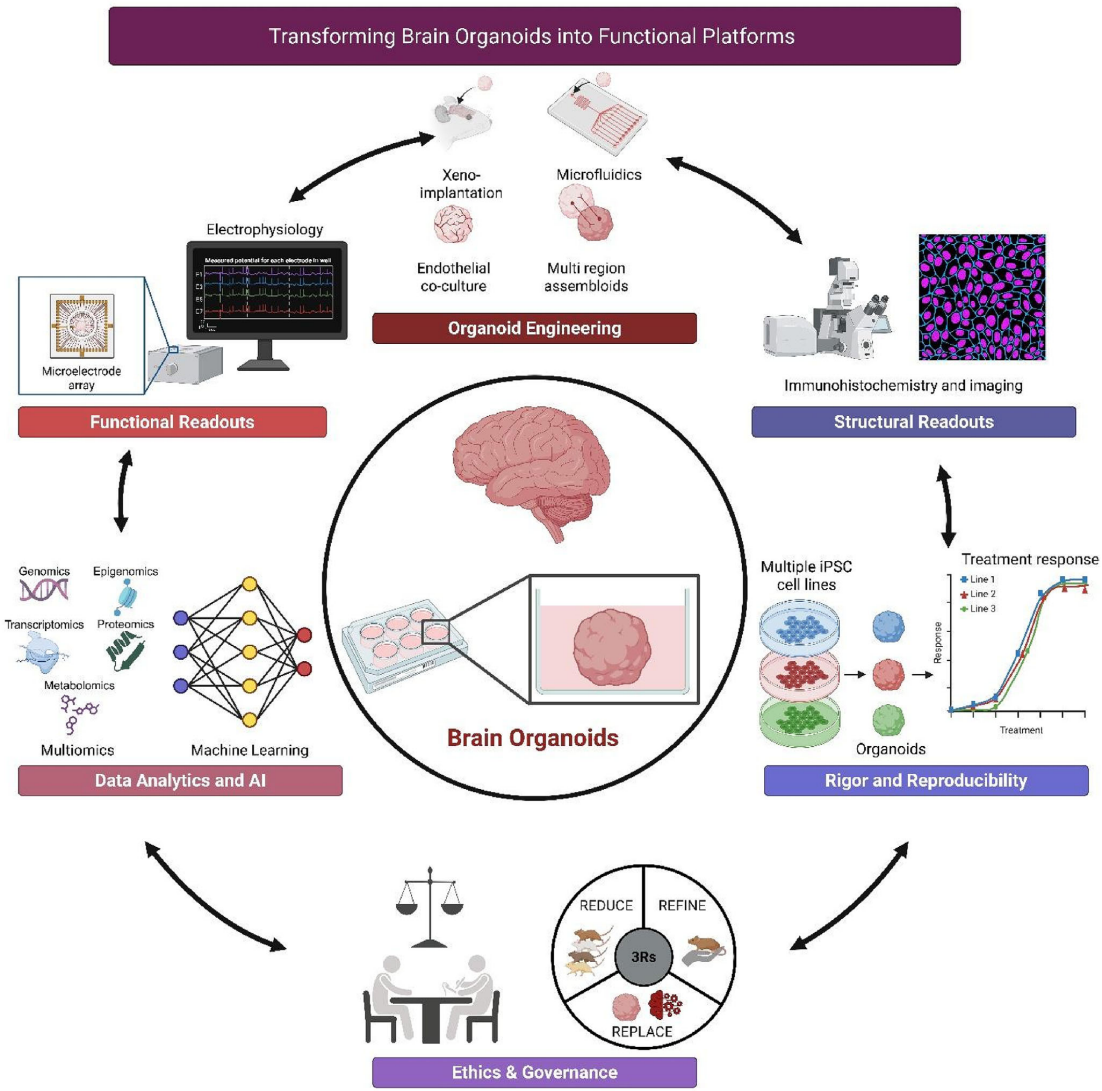
Multidisciplinary Convergence & Transforming Brain Organoids into Functional Platforms: Organoid engineering practices (vascularization, patterning, bio fabrication), functional measurements (electrophysiology, multiomics, etc.), structural analysis (imaging, immunohistochemistry, etc.), data evaluation (machine learning, AI etc.), rigor and reproducibility, as well as appropriate ethical standards, on one side, and the capability to translate models of developmental biology into viable experimental system. Created in BioRender. Pantula, A. (2026) https://BioRender.com/59zibol.

## 2 Organoids as a 3D MPS to Model the Brain

3D MPS provides microscale culture environments that emulate the structural and functional properties of living tissues, enabling the in vitro study of human‐specific physiology and disease [17, 18, 19, 20]. As a result, these organoids have been used to emulate neurodevelopmental [21], neuropsychiatric [22, 23, 24], and neurodegenerative diseases [25, 26]. These models extend beyond disease modeling, serving as human‐relevant platforms to evaluate developmental neurotoxicity [27] induced by drugs, chemicals, and environmental pollutants [28].

Brain organoids derived from pluripotent stem cells provide a self‐organizing three‐dimensional MPS platform that recreates key features of human neurodevelopment, including spatial patterning, cellular diversity, and neuronal specification. However, their ability to model higher‐order brain functions remains constrained by intrinsic size limits, as organoids larger than ∼500–700 µm experience oxygen and nutrient diffusion failure, leading to necrotic cores and structural collapse [29, 30].

To mitigate these constraints, several vascularization strategies have emerged. Xenoimplantation into rodent hosts has enabled host‐derived vascular invasion, promoting long‐term survival and neural differentiation in constructs up to 4 mm in diameter [31, 32, 33]. Yet interspecies differences in endothelial signaling, ethical concerns, and limited scalability hinder this approach. Consequently, attention has shifted to in vitro vascularization, primarily through endothelial co‐culture techniques [34, 35], either via direct co‐culture [36] or within microfluidic well systems to interface the organoid and spatially organize vasculature via perfusion or spinning culture [37, 38]. Despite progress, endothelial co‐culture approaches often yield peripheral, non‐hierarchical vessels with limited penetration into the organoid core, limiting overall oxygenation and size.

To overcome this, convection‐driven microfluidic systems, or organ‐on‐a‐chip devices, have been developed [39] to replace biological vasculature with precisely controlled fluid flow. These strategies use either 3D‐culture areas and channels for culture medium flow and perfusion, micropillars between which cerebral organoids are generated and cultured [40]; and, lastly, a device adapted to a cerebral organoid culture in an air‐liquid interface [41]. These devices use forced convection to deliver nutrients and oxygen uniformly throughout the organoid, thereby sustaining viability and function beyond the limits of static culture [39, 42].

Later innovations, such as high‐throughput perfusion bioreactors and guided‐growth microfluidics, further improved reproducibility and even enabled modeling of cortical folding [43]. While microfluidic systems successfully address nutrient diffusion, they rely on non‐physiological unidirectional flow rather than internal vascular networks, constraining long‐term morphogenesis and 3D expansion beyond 600–700 µm [44]. To address these limitations, recent studies have explored synthetic vascularization via 3D printing, microfabrication, and biomaterials engineering, creating perfusable networks that mimic natural capillary geometry and support large‐scale tissue growth [45, 46, 47]. We summarize all strategies in Table 1.

**TABLE 1:** Comparative summary of organoid engineering strategies.

Strategy	Physiological relevance	Scalability	Reproducibility	Key limitation	Reference
Xenotransplantation / host‐derived vascularization	Provides functional host‐derived perfusion and vascular support within the implanted organoid	Limited by the need for animal implantation and in vivo maintenance	Lower experimental control because vascularization and host integration depend on the recipient environment	Xenogeneic interactions, ethical considerations, and limited throughput/scalability	[31, 32, 33]
Endothelial co‐culture / in vitro vascularization	Introduces human endothelial‐neural interactions and vascular signaling within an entirely in vitro system	Compatible with parallel in vitro culture, but additional cell types, co‐culture steps, and increased media requirements add complexity and cost at scale.	Depends on consistent endothelial differentiation, co‐culture conditions, and vessel organization.	Vessels are often peripheral, non‐hierarchical, and show limited penetration into the organoid core	[34, 35]
Microfluidic perfusion / organ‐on‐chip	Provides controlled delivery of oxygen, nutrients, and soluble cues, improving viability beyond static culture	Amenable to parallelization and higher‐throughput formats, but device fabrication, tubing, and fluid handling can complicate scale‐up	High control over flow rate, geometry, and exposure conditions improves experimental consistency	Channel/device dimensions can limit organoid size; external flow does not replace an internal vascular network.	[39, 42]
Synthetic perfusable vascular networks	Introduces defined perfusable channels that better approximate internal vascular geometry and support transport through larger tissue volumes	Potentially scalable through 3D printing and microfabrication, but fabrication complexity and resolution can limit throughput	Channel geometry and flow conditions can be engineered with relatively high control	While diffusion limitations are addressed, engineered channels may not fully reproduce native vascular hierarchy, endothelial remodeling, or vascular signaling.	[45, 46, 47]
Morphogen‐gradient patterning	Recreates spatial developmental cues to generate region‐specific neural identities and organized tissue domains	Compatible with controlled in vitro systems, but increasing spatial complexity requires more elaborate gradient‐generation platforms	Defined morphogen concentrations and delivery profiles provide relatively high experimental control	Spatial patterning alone may be insufficient to establish long‐range connectivity or functional circuit integration.	[48, 49, 50, 51]
Multi‐region assembloids	Enables interactions between defined brain regions, including cell migration, axonal connectivity, and circuit‐level communication	Modular and extensible, but adding regions increases differentiation, assembly, and culture complexity	Sensitive to fusion efficiency, developmental timing, regional proportions, and alignment between tissues	Anatomical fusion may not necessarily produce reproducible functional connectivity or network synchronization.	m [52, 53, 54]
Multi‐organ assembloids	Incorporates additional neural and non‐neural cell populations to model multicellular interactions absent from conventional organoids	Addition of independently differentiated cell types increases production complexity and handling requirements	Depends on consistent lineage specification, cell ratios, maturation state, and timing of integration	Variable cellular composition, asynchronous maturation, incompatible media requirements, and cross‐lineage contamination can complicate co‐culture.	[55]
Multi‐gradient / multi‐layer assembloids	Recreates spatially organized signaling environments or stratified tissue architecture with greater developmental complexity	Increasing spatial and architectural complexity reduces throughput and complicates assembly	Requires reproducible gradient formation, layer organization, and synchronization across domains	Greater structural complexity does not necessarily translate into physiologically integrated or functional circuitry.	[56, 57]

Collectively, these approaches illustrate a persistent trade‐off between biological fidelity and engineering control: biological vascularization provides endothelial signaling and tissue integration but remains difficult to standardize and scale, whereas engineered perfusion improves transport and experimental control without fully recapitulating the architecture and function of a native vascular network. Thus, establishing reproducible, perfusable human vascular systems that simultaneously support tissue growth, maturation, and vascular signaling remains a central bottleneck.

## 3 From Patterning Organoids to Interconnected Assembloids

While the large‐scale culture of organoids enhances maturation, it cannot reproduce the functional connectivity between distinct brain regions. Human brain development relies on coordinated signaling between distinct progenitor domains and evolving tissue systems, where intrinsic genetic programs intersect with extrinsic cues to guide spatial organization. This interplay establishes region‐specific identities while enabling cross‐domain interactions that ultimately shape integrated brain architecture [58].

Early efforts to spatiotemporally trigger pathways employed osmotic‐pump microfluidic systems to deliver gradients of neural morphogens such as sonic hedgehog (SHH), as well as signaling molecules such as FGF8 and BMP4, directing neural progenitor differentiation at the sub‐millimeter scale [48, 49, 50, 51]. Although constrained in size, these platforms demonstrated the feasibility of generating spatially organized tissues, as shown by neural tube–like patterning induced through precise gradients of purmorphamine (a SHH agonist) and retinoic acid [48, 49, 50, 51].

Taking this forward, the development of assembloids, fused organoids representing different neural domains, also addressed this size gap by enabling circuit‐level modeling of migration, synaptogenesis, and network synchronization. The modular nature of assembloids allows researchers to study cells and brain regions either in isolation or after controlled integration with other organoids, dissecting both cellular phenotypes and region‐specific effects in brain development and disease. Unlike approaches that mix cell lineages in 2D cultures or conventional 3D cultures, assembloids leverage self‐organization to enable complex cell‐cell interactions, circuit formation, and maturation in long‐term cultures [59]. Established protocols for generating assembloids involve differentiating region‐specific organoids from human pluripotent stem cells through sequential pathway modulation, followed by physical fusion under optimized conditions that maintain regional identities while supporting cellular integration.

Current assembloids can be broadly categorized into four types based on their assembly strategies: multi‐region (connecting different brain areas) [52, 53, 54], multi‐lineage (incorporating diverse cell types) [55], multi‐gradient (establishing morphogen gradients) [54], and multilayer (creating stratified tissue architecture) [56, 57], each designed to replicate specific biological phenomena with high fidelity. However, increasing the number of assembled regions does not necessarily produce physiologically integrated circuitry. Fusion efficiency, relative developmental timing, regional proportions, axonal targeting, and network synchronization remain difficult to control across constructs. We summarize all strategies in Table 1. The next challenge is therefore not simply increasing assembloid complexity, but establishing reproducible functional integration between defined neural regions and demonstrating that these connections recapitulate relevant developmental or disease‐associated circuit behavior.

## 4 Imaging and Functional Readouts

### 4.1 Structural Imaging

Imaging brain organoids presents unique technical challenges that must be balanced against the need for comprehensive spatial and temporal information. While advanced techniques such as multi‐photon microscopy [60], three‐photon imaging [61, 62], and planar‐illumination two‐photon microscopy [63] enable deep tissue penetration and reduced phototoxicity, light scattering in dense 3D tissues remains a fundamental limitation, particularly for organoids exceeding 2–3 mm in diameter. The tissue opacity increases with organoid maturation as cellular density and myelination progress, creating a trade‐off between developmental stage and imaging depth.

Label‐free approaches [62, 64, 65] offer non‐invasive longitudinal tracking without the need for genetic modification or exogenous dyes, but often sacrifice molecular specificity compared to fluorescent reporters, making it challenging to identify specific cell types or track particular molecular pathways. Multi‐scale imaging from meso‐ to nanoscale resolution [66] requires correlative workflows that are technically demanding and time‐intensive, often necessitating sample transfer between instruments and careful registration of images acquired at vastly different magnifications. However, sample preparation for high‐resolution imaging often requires tissue clearing protocols that can alter tissue morphology or compromise downstream molecular analyses.

### 4.2 Electrophysiology

A major advance in organoid engineering also lies in functionally characterizing neuronal activity as the organoid is cultured. Electrode‐based microinstrumentation enables direct, high‐resolution monitoring of neural activity beyond the limits of optical assays. Evolving from patch clamping to planar microelectrode arrays (MEAs) to 3D stretchable and mesh architectures [67, 68, 69, 70, 71], these systems enable minimally invasive, long‐term recording of electrical and chemical signaling, providing an integrated platform to study maturation, connectivity, and drug responses in brain organoids. Over the last few years, studies have also demonstrated that neural cultures interfaced with MEAs can exhibit adaptive responses to real‐time environmental feedback, first in two‐dimensional neuronal networks and more recently in three‐dimensional brain organoids subjected to electrical [72] and optical [73] stimulation.

### 4.3 Functional Imaging

Functional imaging modalities such as calcium imaging, which exploit transient increases in intracellular calcium concentration that accompany neuronal firing to visualize network activity in real time [74, 75, 76], provide critical insights into electrophysiological maturation and connectivity patterns within organoids.

However, these approaches are limited by indicator kinetics, photobleaching, expression levels, the challenge of distinguishing true neuronal activity from motion artifacts in spontaneously contracting organoids, and the ongoing challenge of standardizing functional readouts across laboratories [77].

Despite these constraints, combining complementary imaging modalities, including confocal microscopy, multiphoton techniques, and photoacoustic imaging, with computational analysis can provide increasingly comprehensive characterization of organoid development, structure, and function. Together, these structural and functional readouts generate multidimensional datasets, creating a corresponding need for computational frameworks that can integrate information across modalities.

## 5 AI‐Driven Analysis and Multimodal Integration

Machine learning and AI tools are increasingly used to automate image analysis [78], model cellular phenotypes, normalize functional measurements, and identify disease‐associated features and candidate biomarkers [79] from high‐dimensional datasets. For example, longitudinal bright‐field imaging coupled with machine‐learning classification has been used for non‐invasive quality control and pre‐selection of brain organoids based on physiological quality and growth dynamics [80]. In disease modeling, Cheng et al. [24] applied machine‐learning classification to electrophysiological recordings from patient‐derived cerebral organoids to distinguish control, schizophrenia, and bipolar disorder phenotypes, with classification performance improving following electrical stimulation.

These approaches can reduce dependence on subjective assessment and extract quantitative phenotypes from high‐dimensional structural and functional measurements. However, they introduce computational complexity and require substantial data storage infrastructure, with a single multi‐day time‐lapse experiment potentially generating terabytes of raw image data.

Beyond automated analysis of individual readouts, an emerging challenge is integrating multimodal organoid datasets. As complementary datasets become increasingly high‐throughput, they are still commonly generated and analyzed through separate workflows, limiting the ability to relate molecular, structural, and electrophysiological changes within the same system. This is particularly important in pharmacological studies, where treatment effects may differ across transcriptional, cellular, structural, and functional scales. A unified analytical framework is therefore needed to determine whether molecular perturbations translate into meaningful changes in tissue organization and circuit function.

Machine‐learning approaches offer a route to bridge these data silos by learning shared representations across high‐dimensional modalities, identifying cross‐modal features associated with functional phenotypes, and relating integrated molecular signatures to treatment response. Deep generative models can already integrate paired and unpaired transcriptomic, epigenomic, and proteomic measurements while accounting for batch effects and missing modalities [81]. In organoid systems, machine‐learning frameworks have linked transcriptomic programs to pharmacological sensitivity and predicted treatment responses, while recent multi‐omic organoid studies have integrated genetic, transcriptomic, miRNA, and pharmacological datasets to construct drug‐response models [82, 83]. Such approaches could shift organoid pharmacology from parallel, modality‐specific readouts toward integrated response profiles that relate molecular perturbations to functional outcomes. Extending these approaches to functional readouts such as electrophysiology and calcium imaging could further link molecular responses to changes in network activity and circuit dynamics, providing a more complete assessment of whether pharmacological perturbations produce functionally meaningful effects.

These computational frameworks could also support comparison with external biological reference datasets, including animal models, by identifying conserved and divergent molecular and functional signatures across species. Such cross‐species integration may help position organoid‐derived responses within the broader preclinical evidence base while highlighting human‐specific phenotypes.

More broadly, the value of such integrated organoid models will depend on whether multimodal response signatures are reproducible, interpretable, and predictive across experimental systems. This becomes particularly relevant as organoid and computational models are incorporated into new approach methodology (NAM) frameworks for nonclinical drug development [84], where context‐of‐use validation and quantitative evidence of predictive performance will be essential for translational adoption.

## 6 Reproducibility, Rigor, and Ethics

Human brain organoids offer unprecedented opportunities for modeling development and disease; however, the field must first address significant challenges in reproducibility and experimental rigor to fully realize this potential in a high‐throughput translational setting [85].

### 6.1 Reproducibility

One of the biggest challenges in organoid engineering is creating replicable, reproducible culture models. Variability in stem cell source, culture conditions, and protocol execution produces pronounced morphological and transcriptional heterogeneity, complicating robust comparisons both within and between experiments. Even under established procedures [86, 87, 88], individual organoids and batches often develop distinct tissue architectures and cellular compositions, limiting their ability to serve as reliable disease models or screening platforms.

Recent research has sought to mitigate these issues through technological and engineering innovations, including advanced bioreactor systems and optimized culture environments, thereby reducing batch‐to‐batch variability and promoting consistent maturation. Protocol refinements incorporating mechanical control, aggregation phase optimization, and metabolic profiling have improved both structural and functional stability across diverse genetic backgrounds. Nonetheless, unresolved challenges remain, particularly with asynchronous tissue maturation, metabolic stress, and insufficient vascularization, which constrain the physiological maturity and translational relevance of long‐term cultures [28, 89, 90].

### 6.2 Rigor

Current methods for characterizing organoids are often limited by a lack of standards and reliance on subjective assessments, ultimately hindering their widespread application [91]. A critical first step is defining the tissue‐specific critical quality attributes (CQAs [86, 92, 93]), such as morphology, cellular composition, network activity, structural maturation, metabolic function, and biomechanical properties; these must be quantified, along with acceptable ranges that would support regulatory decision‐making. Establishing these benchmarks will also require physiologically relevant reference materials and tissue mimics [94], as well as metrologically traceable [95] characterization methods and orthogonal validation across independent measurements. At the model level, initiatives such as the NIH Standardized Organoid Modeling (SOM) Center are similarly developing standardized, fit‐for‐purpose organoid protocols and benchmarking frameworks to improve reproducibility and comparability across laboratories [96].

These measurement priorities are increasingly aligned with broader regulatory efforts to advance human‐relevant New Approach Methodologies (NAMs). In the United States, recent FDA and legislative initiatives support greater use of human‐based experimental systems and computational approaches in nonclinical drug development [97, 98]. European Commission efforts similarly aim to accelerate the transition toward non‐animal methods, particularly within chemical safety assessment [99]. Internationally, Japan's PMDA has established activities focused specifically on NAMs, alongside longstanding Japanese contributions to OECD non‐animal Test Guidelines [100, 101]. South Korea [102, 103], China [104], and Australia (NHMRC [105] and CSIRO [106]) have continued to expand regulatory acceptance of alternative testing approaches.

Beyond these model‐ and regulatory‐level benchmarks, validation should also extend beyond the organoid system itself. Cross‐species comparisons can determine which molecular and functional phenotypes are conserved between organoid systems and established animal models and which may reflect human‐specific biology [107, 108, 109]. Comparing control and disease‐associated signatures across species could therefore contextualize organoid phenotypes within the existing preclinical evidence base while identifying areas in which human‐derived models provide complementary or divergent information. Such comparisons will require careful consideration of ortholog mapping, developmental stage, cell‐type composition, and species‐specific physiology [108, 109, 110].

Harmonized, transparent data‐analysis pipelines are also needed to ensure accuracy, minimize artifacts, and generate auditable, metadata‐rich datasets. By establishing this comprehensive framework for measurement, validation, and data governance, we can create a foundation for scientific reliability, clinical relevance, and regulatory confidence in organoid‐based platforms, ultimately accelerating innovation and breakthroughs in the field. However, technical standardization and validation alone are not sufficient for responsible translation. As brain organoids become increasingly complex and are deployed in new experimental and translational contexts, their development must also be accompanied by appropriate ethical and governance frameworks.

### 6.3 Ethics

Current ethical considerations surrounding human brain organoids primarily concern donor consent and privacy, ownership and commercialization, and appropriate research oversight. Regulatory frameworks for human brain organoid research remain limited and vary internationally; most guidelines, such as those from the International Society for Stem Cell Research, treat organoids as in vitro models exempt from specialized oversight, given the current lack of evidence for consciousness or sentience [111]. Human brain organoid research raises complex consent challenges, as traditional anonymized or broad consent models may not suit studies using identifiable donor tissues or evolving applications; thus, researchers are exploring dynamic, tiered, or governance‐based consent frameworks to balance donor rights, privacy, and scientific flexibility [112, 113, 114].

Commercialization introduces additional ethical questions surrounding ownership, profit, and donor rights. Although donated biological material has traditionally been treated as an altruistic contribution to research, increasingly complex organoid applications raise questions regarding benefit‐sharing, commercial use, and corporate accountability. Clear governance regarding ownership and commercialization will therefore be important as organoid technologies move toward broader translational use.

Prospective ethical concerns may arise as brain organoids gain greater structural and functional complexity or are incorporated into more sophisticated experimental platforms. Questions of moral status, including the potential for pain perception, consciousness, or self‐awareness, therefore remain an important area of ethical consideration as the technology develops [115]. These questions may become particularly relevant as neural organoids are incorporated into biohybrid or closed‐loop systems. Neural cultures have already demonstrated functional responses to environmental feedback [116], establishing the technical basis for systems in which neural activity can dynamically regulate stimulation or external devices.

Importantly, there is currently no evidence that human brain organoids are conscious. Nevertheless, as organoids become more anatomically and physiologically complex, assessing the possibility of consciousness and its ethical implications remains an important consideration for future research [117]. For xenotransplantation studies, the principles of Replace, Reduce, and Refine (3Rs) remain relevant to minimizing animal use and harm while maintaining scientific necessity [118, 119]. Overall, given the dynamic and uncertain nature of organoid technologies, a framework of mindful innovation, one that continuously adapts ethical oversight to technological progress, may offer a more flexible and comprehensive approach to future governance [120].

## 7 Conclusion

Brain organoids now stand at the intersection of biology, engineering, and ethics, evolving from static culture systems into dynamic, responsive models that capture facets of human brain function. Adopting a framework of mindful innovation, one that couples scientific ambition with ethical adaptability, will be key to guiding this field toward meaningful translational impact while safeguarding the values that define responsible science. Their continued advancement and clinical translation will depend on parallel progress in reproducibility, standards development, technological integration, and responsible governance.

## Conflicts of Interest

The authors declare no conflicts of interest.

## Data Availability

The authors have nothing to report.

## References

[1] L. Whitmore and D. Beck , “Current Challenges and Future Directions for Brain Age Prediction in Children and Adolescents,” Nature Communications 16, no. 1 (2025): 7771, 10.1038/s41467-025-63222-7. PMC12368027 40835837

[2] E. Dessaud , L. L. Yang , K. Hill , et al., “Interpretation of the Sonic Hedgehog Morphogen Gradient by a Temporal Adaptation Mechanism,” Nature 450, no. 7170 (2007): 717–720, 10.1038/nature06347. 18046410

[3] T. J. Nowakowski , A. Bhaduri , A. A. Pollen , et al., “Spatiotemporal Gene Expression Trajectories Reveal Developmental Hierarchies of the human Cortex,” Science 358, no. 6368 (2017): 1318–1323, 10.1126/science.aap8809. PMC5991609 29217575

[4] S. C. Noctor , A. C. Flint , T. A. Weissman , R. S. Dammerman , and A. R. Kriegstein , “Neurons Derived from Radial Glial Cells Establish Radial Units in Neocortex,” Nature 409, no. 6821 (2001): 714–720, 10.1038/35055553. 11217860

[5] R. C. Paolicelli , G. Bolasco , F. Pagani , et al., “Synaptic Pruning by Microglia Is Necessary for Normal Brain Development,” Science 333, no. 6048 (2011): 1456–1458, 10.1126/science.1202529. 21778362

[6] E. M. Gibson , D. Purger , C. W. Mount , et al., “Neuronal Activity Promotes Oligodendrogenesis and Adaptive Myelination in the Mammalian Brain,” Science 344, no. 6183 (2014): 1252304, 10.1126/science.1252304. PMC4096908 24727982

[7] R. D. Hodge , T. E. Bakken , J. A. Miller , et al., “Conserved Cell Types with Divergent Features in human versus Mouse Cortex,” Nature 573, no. 7772 (2019): 61–68, 10.1038/s41586-019-1506-7. PMC6919571 31435019

[8] J. C. Silbereis , S. Pochareddy , Y. Zhu , M. Li , and N. Sestan , “The Cellular and Molecular Landscapes of the Developing Human Central Nervous System,” Neuron 89, no. 2 (2016): 248–268, 10.1016/j.neuron.2015.12.008. PMC4959909 26796689

[9] J. F. Cryan and A. Holmes , “The Ascent of Mouse: Advances in Modelling human Depression and Anxiety,” Nature Reviews Drug Discovery 4, no. 9 (2005): 775–790, 10.1038/nrd1825. 16138108

[10] X. Zhao and A. Bhattacharyya , “Human Models Are Needed for Studying Human Neurodevelopmental Disorders,” The American Journal of Human Genetics 103, no. 6 (2018): 829–857, 10.1016/j.ajhg.2018.10.009. PMC6288051 30526865

[11] “FDA Announces Plan to Phase out Animal Testing Requirement for Monoclonal Antibodies and Other Drugs,” can be found under https://www.fda.gov/news‐events/press‐announcements/fda‐announces‐plan‐phase‐out‐animal‐testing‐requirement‐monoclonal‐antibodies‐and‐other‐drugs, 2025.

[12] J. J. Han , “FDA Modernization Act 2.0 Allows for Alternatives to Animal Testing,” Artificial Organs 47, no. 3 (2023): 449–450, 10.1111/aor.14503. 36762462

[13] K. Takahashi and S. Yamanaka , “Induction of Pluripotent Stem Cells from Mouse Embryonic and Adult Fibroblast Cultures by Defined Factors,” Cell 126, no. 4 (2006): 663–676, 10.1016/j.cell.2006.07.024. 16904174

[14] E. Suarez‐Martinez , I. Suazo‐Sanchez , M. Celis‐Romero , and A. Carnero , “3D and Organoid Culture in Research: Physiology, Hereditary Genetic Diseases and Cancer,” Cell & Bioscience 12, no. 1 (2022): 39, 10.1186/s13578-022-00775-w. PMC8973959 35365227

[15] D. Lullo and E. Kriegstein , “The Use of Brain Organoids to Investigate Neural Development and Disease,” Nature Reviews Neuroscience 18, no. 10 (2017): 573–584, 10.1038/nrn.2017.107. PMC5667942 28878372

[16] C. Solana‐Manrique , A. M. Sánchez‐Pérez , N. Paricio , and S. Muñoz‐Descalzo , “Two‐ and Three‐Dimensional In Vitro Models of Parkinson's and Alzheimer's Diseases: State‐of‐the‐Art and Applications,” International Journal of Molecular Sciences 26 (2025): 620, 10.3390/ijms26020620. PMC11766061 39859333

[17] M. A. Lancaster and J. A. Knoblich , “Generation of Cerebral Organoids from human Pluripotent Stem Cells,” Nature Protocols 9, no. 10 (2014): 2329–2340, 10.1038/nprot.2014.158. PMC4160653 25188634

[18] M. Simian and M. J. Bissell , “Organoids: A Historical Perspective of Thinking in Three Dimensions,” Journal of Cell Biology 216, no. 1 (2017): 31–40, 10.1083/jcb.201610056. PMC5223613 28031422

[19] O. Commissioner , “About Alternative Methods,” can be found under https://www.fda.gov/science‐research/advancing‐alternative‐methods‐fda/about‐alternative‐methods, 2024.

[20] T. Kadoshima , H. Sakaguchi , T. Nakano , et al., “Self‐Organization of Axial Polarity, inside‐Out Layer Pattern, and Species‐Specific Progenitor Dynamics in human ES Cell–derived Neocortex,” Proceedings of the National Academy of Sciences 110, no. 50 (2013): 20284–20289, 10.1073/pnas.1315710110. PMC3864329 24277810

[21] C. A. Trujillo and A. R. Muotri , “Brain Organoids and the Study of Neurodevelopment,” Trends in Molecular Medicine 24, no. 12 (2018): 982–990, 10.1016/j.molmed.2018.09.005. PMC6289846 30377071

[22] Y. Sun and W. Pan , “Brain Organoids: A New Paradigm for Studying Human Neuropsychiatric Disorders,” Frontiers in Neuroscience 19 (2025): 1699814, 10.3389/fnins.2025.1699814. PMC12620469 41255817

[23] A. Kathuria , K. Lopez‐Lengowski , S. S. Jagtap , et al., “Transcriptomic Landscape and Functional Characterization of Induced Pluripotent Stem Cell–Derived Cerebral Organoids in Schizophrenia,” JAMA Psychiatry 77, no. 7 (2020): 745, 10.1001/jamapsychiatry.2020.0196. PMC7081156 32186681

[24] K. Cheng , A. Williams , A. Kshirsagar , et al., “Machine Learning‐Enabled Detection of Electrophysiological Signatures in iPSC‐Derived Models of Schizophrenia and Bipolar Disorder,” APL Bioengineering 9, no. 3 (2025): 036118, 10.1063/5.0250559. PMC12456967 40995147

[25] S. Shaikh , L. Siddique , H. T. Khalifey , et al., “Brain Organoid Model Systems of Neurodegenerative Diseases: Recent Progress and Future Prospects,” Frontiers in Neuroscience 19 (2025): 1604435, 10.3389/fnins.2025.1604435. PMC12141293 40486729

[26] D. Pamies , P. Barreras , K. Block , et al., “A human Brain Microphysiological System Derived from Induced Pluripotent Stem Cells to Study Neurological Diseases and Toxicity,” Altex 34 (2017): 362–376, 10.14573/altex.1609122. PMC6047513 27883356

[27] Y. Cao , “The uses of 3D human brain organoids for neurotoxicity evaluations: A review,” Neurotoxicology 91 (2022): 84–93, 10.1016/j.neuro.2022.05.004. 35561940

[28] H. Castiglione , L. Madrange , C. Baquerre , et al., “Towards a Quality Control Framework for Cerebral Cortical Organoids,” Scientific Reports 15, no. 1 (2025): 29431, 10.1038/s41598-025-14425-x. PMC12340003 40790335

[29] X. Qian , H. Song , and G.‐L. Ming , “Brain Organoids: Advances, Applications and Challenges,” Development 146 (2019): dev166074, 10.1242/dev.166074. PMC6503989 30992274

[30] N. Urrestizala‐Arenaza , S. Cerchio , F. Cavaliere , and C. Magliaro , “Limitations of human Brain Organoids to Study Neurodegenerative Diseases: A Manual to Survive,” Frontiers in Cellular Neuroscience 18 (2024): 1419526, 10.3389/fncel.2024.1419526. PMC11267621 39049825

[31] N. Daviaud , R. H. Friedel , and H. Zou , “Vascularization and Engraftment of Transplanted Human Cerebral Organoids in Mouse Cortex,” eNeuro 5, no. 6 (2018): ENEURO0219, 10.1523/ENEURO.0219-18.2018. PMC6243198 30460331

[32] A. A. Mansour , J. T. Gonçalves , C. W. Bloyd , et al., “An in Vivo Model of Functional and Vascularized human Brain Organoids,” Nature Biotechnology 36, no. 5 (2018): 432–441, 10.1038/nbt.4127. PMC6331203 29658944

[33] Y. Shi , L. Sun , M. Wang , et al., “Vascularized human Cortical Organoids (vOrganoids) Model Cortical Development in Vivo,” PLoS Biology 18, no. 5 (2020): 3000705, 10.1371/journal.pbio.3000705. PMC7250475 32401820

[34] S. Zhang , Z. Wan , and R. D. Kamm , “Vascularized Organoids on a Chip: Strategies for Engineering Organoids with Functional Vasculature,” Lab on a Chip 21, no. 3 (2021): 473–488, 10.1039/D0LC01186J. PMC8283929 33480945

[35] E. Zahmatkesh , N. Khoshdel‐Rad , H. Mirzaei , et al., “Evolution of Organoid Technology: Lessons Learnt in Co‐Culture Systems from Developmental Biology,” Developmental Biology 475 (2021): 37–53, 10.1016/j.ydbio.2021.03.001. 33684433

[36] G. D. Vatine , R. Barrile , M. J. Workman , et al., “Human iPSC‐Derived Blood‐Brain Barrier Chips Enable Disease Modeling and Personalized Medicine Applications,” Cell Stem Cell 24, no. 6 (2019): 995–1005.e6, 10.1016/j.stem.2019.05.011. 31173718

[37] N. Shin , Y. Kim , J. Ko , et al., “Vascularization of iNSC Spheroid in a 3D Spheroid‐on‐a‐Chip Platform Enhances Neural Maturation,” Biotechnology and Bioengineering 119, no. 2 (2022): 566–574, 10.1002/bit.27978. PMC9298365 34716703

[38] I. Salmon , S. Grebenyuk , A. R. Abdel Fattah , et al., “Engineering Neurovascular Organoids with 3D Printed Microfluidic Chips,” Lab on a Chip 22, no. 8 (2022): 1615–1629, 10.1039/D1LC00535A. 35333271

[39] Y. Wang , L. Wang , Y. Guo , Y. Zhu , and J. Qin , “Engineering Stem Cell‐Derived 3D Brain Organoids in a Perfusable Organ‐on‐a‐Chip System,” RSC Advances 8, no. 3 (2018): 1677–1685, 10.1039/C7RA11714K. PMC9077091 35540867

[40] Y. Zhu , L. Wang , H. Yu , et al., “In Situ Generation of human Brain Organoids on a Micropillar Array,” Lab on a Chip 17, no. 17 (2017): 2941–2950, 10.1039/C7LC00682A. 28752164

[41] Z. Ao , H. Cai , D. J. Havert , et al., “One‐Stop Microfluidic Assembly of Human Brain Organoids To Model Prenatal Cannabis Exposure,” Analytical Chemistry 92, no. 6 (2020): 4630–4638, 10.1021/acs.analchem.0c00205. 32070103

[42] H. Andersson and A. van den Berg , “Microfabrication and Microfluidics for Tissue Engineering: State of the Art and Future Opportunities,” Lab on a Chip 4, no. 2 (2004): 98, 10.1039/b314469k. 15052347

[43] E. Karzbrun , A. Kshirsagar , S. R. Cohen , J. H. Hanna , and O. Reiner , “Human Brain Organoids on a Chip Reveal the Physics of Folding,” Nature Physics 14, no. 5 (2018): 515–522, 10.1038/s41567-018-0046-7. PMC5947782 29760764

[44] A. Pantula , B. Zhou , I. E. Morales Pantoja , et al., “Computational Modeling of Necrosis in Neural Organoids,” Bioengineering 20 (2025): 044101.

[45] S. Grebenyuk , A. R. Abdel Fattah , M. Kumar , et al., “Large‐Scale Perfused Tissues via Synthetic 3D Soft Microfluidics,” Nature Communications 14, no. 1 (2023): 193, 10.1038/s41467-022-35619-1. PMC9837048 36635264

[46] M. A. Skylar‐Scott , S. G. M. Uzel , L. L. Nam , et al., “Biomanufacturing of Organ‐Specific Tissues With High Cellular Density and Embedded Vascular Channels,” Science Advances 5 (2019): aaw2459, 10.1126/sciadv.aaw2459. PMC6731072 31523707

[47] S. Kim , H. Lee , M. Chung , and N. L. Jeon , “Engineering of Functional, Perfusable 3D Microvascular Networks on a Chip,” Lab on a Chip 13, no. 8 (2013): 1489, 10.1039/c3lc41320a. 23440068

[48] J. Y. Park , S.‐K. Kim , D.‐H. Woo , E.‐J. Lee , J.‐H. Kim , and S.‐H. Lee , “Differentiation of Neural Progenitor Cells in a Microfluidic Chip‐Generated Cytokine Gradient,” Stem Cells 27, no. 11 (2009): 2646–2654, 10.1002/stem.202. 19711444

[49] B. G. Chung , L. A. Flanagan , S. W. Rhee , et al., “Human Neural Stem Cell Growth and Differentiation in a Gradient‐Generating Microfluidic Device,” Lab on a Chip 5, no. 4 (2005): 401, 10.1039/b417651k. 15791337

[50] C. J. Demers , P. Soundararajan , P. Chennampally , et al., “Development‐on‐Chip: In Vitro Neural Tube Patterning with a Microfluidic Device,” Development 143, no. 11 (2016): 1884–1892, 10.1242/dev.126847. PMC4920155 27246712

[51] Y.‐C. Tsai , H. Ozaki , A. Morikawa , et al., “Proof of Concept for Brain Organoid‐on‐a‐Chip to Create Multiple Domains in Forebrain Organoids,” RSC Advances 15 (2025): 3749–3755. 10.1039/d4ra04194a PMC11797153 39911547

[52] J.‐I. Kim , K. Imaizumi , O. Jurjuț , et al., “Human Assembloid Model of the Ascending Neural Sensory Pathway,” Nature 642, no. 8066 (2025): 143–153, 10.1038/s41586-025-08808-3. PMC12137141 40205039

[53] J.‐I. Kim , Y. Miura , M.‐Y. Li , et al., “Human Assembloids Reveal the Consequences of CACNA1G Gene Variants in the Thalamocortical Pathway,” Neuron 112, no. 24 (2024): 4048–4059.e7, 10.1016/j.neuron.2024.09.020. 39419023

[54] S.‐R. Wu and T. J. Nowakowski , “Exploring human Brain Development and Disease Using Assembloids,” Neuron 113, no. 8 (2025): 1133–1150, 10.1016/j.neuron.2025.02.010. PMC12022838 40107269

[55] A. Kshirsagar , H. Mnatsakanyan , S. Kulkarni , et al., “Multi‐Region Brain Organoids Integrating Cerebral, Mid‐Hindbrain, and Endothelial Systems,” Advanced Science (Weinh) 12 (2025): 03768. 10.1002/advs.202503768 PMC12412538 40625223

[56] X. Qian , Y. Su , C. D. Adam , et al., “Sliced Human Cortical Organoids for Modeling Distinct Cortical Layer Formation,” Cell Stem Cell 26, no. 5 (2020): 766–781.e9, 10.1016/j.stem.2020.02.002. PMC7366517 32142682

[57] Y. Wang , S. Chiola , G. Yang , et al., “Modeling human Telencephalic Development and Autism‐Associated SHANK3 Deficiency Using Organoids Generated from Single Neural Rosettes,” Nature Communications 13, no. 1 (2022): 5688, 10.1038/s41467-022-33364-z. PMC9537523 36202854

[58] M. Barber , W. D. Andrews , F. Memi , et al., “Vascular‐Derived Vegfa Promotes Cortical Interneuron Migration and Proximity to the Vasculature in the Developing Forebrain,” Cerebral Cortex 28, no. 7 (2018): 2577–2593, 10.1093/cercor/bhy082. PMC5998991 29901792

[59] Y. Miura , M.‐Y. Li , O. Revah , S.‐J. Yoon , G. Narazaki , and S. P. Pașca , “Engineering Brain Assembloids to Interrogate human Neural Circuits,” Nature Protocols 17, no. 1 (2022): 15–35, 10.1038/s41596-021-00632-z. 34992269

[60] M. E. Lacin and M. Yildirim , “Applications of Multiphoton Microscopy in Imaging Cerebral and Retinal Organoids,” Frontiers in Neuroscience 18 (2024): 1360482, 10.3389/fnins.2024.1360482. PMC10948410 38505776

[61] M. Yildirim , C. Delepine , D. Feldman , et al., “Label‐Free Three‐Photon Imaging of Intact human Cerebral Organoids for Tracking Early Events in Brain Development and Deficits in Rett Syndrome,” Elife 11 (2022): 78079, 10.7554/eLife.78079. PMC9337854 35904330

[62] T. Osaki , W. D. Lee , X. Zhang , et al., “Multi‐Photon, Label‐Free Photoacoustic and Optical Imaging of NADH in Brain Cells,” Light: Science & Applications 14 (2025): 264. 10.1038/s41377-025-01895-x PMC12331929 40775235

[63] I. Rakotoson , B. Delhomme , P. Djian , et al., “Fast 3‐D Imaging of Brain Organoids with a New Single‐Objective Planar‐Illumination Two‐Photon Microscope,” Frontiers in Neuroanatomy 13 (2019): 77, 10.3389/fnana.2019.00077. PMC6710410 31481880

[64] C. E. Serafini , S. Charles , P. Casteleiro Costa , et al., “Non‐Invasive Label‐Free Imaging Analysis Pipeline for in Situ Characterization of 3D Brain Organoids,” Scientific Reports 14, no. 1 (2024): 22331, 10.1038/s41598-024-72038-2. PMC11436713 39333572

[65] B. Wang , R. Ganjee , I. Khandaker , et al., “Deep Learning Based Characterization of human Organoids Using Optical Coherence Tomography,” Biomedical Optics Express 15, no. 5 (2024): 3112, 10.1364/BOE.515781. PMC11161340 38855657

[66] J. E. Rodriguez‐Gatica , V. Iefremova , L. Sokhranyaeva , et al., “Imaging Three‐Dimensional Brain Organoid Architecture From Meso‐ to Nanoscale Across Development,” Development 149 (2022): dev200439, 10.1242/dev.200439. 35899577

[67] Q. Huang , B. Tang , J. C. Romero , et al., “Shell Microelectrode Arrays (MEAs) for Brain Organoids,” Science Advances 8, no. 33 (2022): abq5031, 10.1126/sciadv.abq5031. PMC9385157 35977026

[68] Y. Park , C. K. Franz , H. Ryu , et al., “Three‐Dimensional, Multifunctional Neural Interfaces for Cortical Spheroids and Engineered Assembloids,” Science Advances 7 (2021): abf9153. 10.1126/sciadv.abf9153 PMC7968849 33731359

[69] D. Patel , S. Shetty , C. Acha , et al., “Microinstrumentation for Brain Organoids,” Advanced Healthcare Materials 13, no. 21 (2024): 2302456, 10.1002/adhm.202302456. 38217546

[70] C. A. Thomas Jr , P. A. Springer , G. E. Loeb , Y. Berwald‐Netter , and L. M. Okun , “A Miniature Microelectrode Array to Monitor the Bioelectric Activity of Cultured Cells,” Experimental Cell Research 74, no. 1 (1972): 61–66, 10.1016/0014-4827(72)90481-8. 4672477

[71] A. L. Hodgkin and A. F. Huxley , “A Quantitative Description of Membrane Current and Its Application to Conduction and Excitation in Nerve,” The Journal of Physiology 117, no. 4 (1952): 500–544, 10.1113/jphysiol.1952.sp004764. PMC1392413 12991237

[72] C. Acha , D. George , L. C. Diaz , et al., “Neuromodulation in Neural Organoids with Shell MEAs,” bioRxivorg (2025): 2025, 10.1101/2025.02.18.637712. 41502138

[73] E. Molokanova , T. Zhou , P. Vasupal , et al., “Non‐Genetic Neuromodulation with Graphene Optoelectronic Actuators for Disease Models, Stem Cell Maturation, and Biohybrid Robotics,” Nature Communications 16, no. 1 (2025): 7499, 10.1038/s41467-025-62637-6. PMC12368249 40835596

[74] K. Voitiuk , S. T. Seiler , M. P. de Melo , et al., “A Feedback‐Driven Brain Organoid Platform Enables Automated Maintenance and High‐Resolution Neural Activity Monitoring,” Internet of Things 33 (2025): 101671, 10.1016/j.iot.2025.101671. PMC12781996 41522195

[75] X. He , Y. Wang , Y. Gao , et al., “CalciumZero: A Toolbox for Fluorescence Calcium Imaging on iPSC Derived Brain Organoids,” Brain Informatics 12, no. 1 (2025): 2, 10.1186/s40708-024-00248-5. PMC11746984 39833639

[76] J. Yin and A. M. VanDongen , “Enhanced Neuronal Activity and Asynchronous Calcium Transients Revealed in a 3D Organoid Model of Alzheimer's Disease,” ACS Biomaterials Science & Engineering 7, no. 1 (2021): 254–264, 10.1021/acsbiomaterials.0c01583. 33347288

[77] M. K. Oliva , J. Bourke , D. Kornienko , et al., “Standardizing a Method for Functional Assessment of Neural Networks in Brain Organoids,” Journal of Neuroscience Methods 409 (2024): 110178, 10.1016/j.jneumeth.2024.110178. 38825241

[78] L. Bai , Y. Wu , G. Li , W. Zhang , H. Zhang , and J. Su , “AI‐Enabled Organoids: Construction, Analysis, and Application,” Bioactive Materials 31 (2024): 525. 10.1016/j.bioactmat.2023.09.005 PMC10511344 37746662

[79] J. Betge , N. Rindtorff , J. Sauer , et al., “The Drug‐Induced Phenotypic Landscape of Colorectal Cancer Organoids,” Nature Communications 13, no. 1 (2022): 3135, 10.1038/s41467-022-30722-9. PMC9170716 35668108

[80] S. Charles , E. Jackson‐Holmes , G. Sun , et al., “Non‐Invasive Quality Control of Organoid Cultures Using Mesofluidic CSTR Bioreactors and High‐Content Imaging,” Advanced Materials Technologies 10, no. 3 (2025): 2400473, 10.1002/admt.202400473. PMC12002419 40248044

[81] T. Ashuach , M. I. Gabitto , R. V. Koodli , G.‐A. Saldi , M. I. Jordan , and N. Yosef , “MultiVI: Deep Generative Model for the Integration of Multimodal Data,” Nature Methods 20, no. 8 (2023): 1222–1231, 10.1038/s41592-023-01909-9. PMC10406609 37386189

[82] J. Kong , H. Lee , D. Kim , et al., “Network‐Based Machine Learning in Colorectal and Bladder Organoid Models Predicts Anti‐Cancer Drug Efficacy in Patients,” Nature Communications 11, no. 1 (2020): 5485, 10.1038/s41467-020-19313-8. PMC7599252 33127883

[83] C.‐Y. Kim , S.‐C. Kim , R. Shin , et al., “Integrative Multi‐Omics of Matched Primary and Liver Metastatic Colorectal Cancer Organoids Identifies Putative Molecular Targets,” Translational Oncology 63 (2026): 102592, 10.1016/j.tranon.2025.102592. PMC12637260 41192148

[84] Center for Drug Evaluation, Research , “General Considerations for the Use of New Approach Methodologies in Drug Development,” can be found under https://www.fda.gov/regulatory‐information/search‐fda‐guidance‐documents/general‐considerations‐use‐new‐approach‐methodologies‐drug‐development, 2026.

[85] S.‐H. Kim and M.‐Y. Chang , “Application of Human Brain Organoids—Opportunities and Challenges in Modeling Human Brain Development and Neurodevelopmental Diseases,” International Journal of Molecular Sciences 24, no. 15 (2023): 12528, 10.3390/ijms241512528. PMC10420018 37569905

[86] H. Lee , S. Yang , K. J. Lee , et al., “Standardization and Quality Assessment for human Intestinal Organoids,” Frontiers in Cell and Developmental Biology 12 (2024): 1383893, 10.3389/fcell.2024.1383893. PMC11424408 39329062

[87] T. O. F. Olivatti , G. P. Alcantara , A. C. C. E. Lemos , M. G. da Silva , and H. A. Miot , “Standardization of Organoid Culture for Evaluation of Melanogenesis Induced by UVB, UVA and Visible Light,” Anais Brasileiros de Dermatologia 95, no. 1 (2020): 46–51, 10.1016/j.abd.2019.06.005. PMC7058865 31901368

[88] O. Krefft , A. Jabali , V. Iefremova , P. Koch , and J. Ladewig , “Generation of Standardized and Reproducible Forebrain‐Type Cerebral Organoids from Human Induced Pluripotent Stem Cells,” Journal of visualized experiments: JoVE (2018): 1–8, 10.3791/56768. PMC5908685 29443048

[89] S. O. Sandoval , G. Cappuccio , K. Kruth , et al., “Rigor and Reproducibility in Human Brain Organoid Research,” Stem Cell Reports 19 (2024): 796–816. 10.1016/j.stemcr.2024.04.008 PMC11297560 38759644

[90] I. Faravelli , N. Antón‐Bolaños , J. R. Brown , and P. Arlotta , “Brain Organoids: Tools for Understanding the Uniqueness and Individual Variability of the Human Brain,” Annual Review of Genomics and Human Genetics 26, no. 1 (2025): 299–320, 10.1146/annurev-genom-111522-014009. 40854826

[91] H. Castiglione , L. Madrange , C. Baquerre , et al., “Towards a Quality Control Framework for Cerebral Cortical Organoids,” Neuroscience 15 (2025): 29431. 10.1038/s41598-025-14425-x PMC12340003 40790335

[92] A. da H Alves , N. M. Ennes do Valle , B. Y. Yokota‐Moreno , et al., “Stem Cell‐Derived Neural Organoids as Platforms to Investigate Glioblastoma Invasion and Migration: A Systematic Review,” World Journal of Stem Cells 17 (2025): 108898. 10.4252/wjsc.v17.i8.108898 PMC12427015 40951702

[93] U. S. F. A. D. Administration Guidance for Industry: Q8(R2) Pharmaceutical Development , (Silver Spring, 2009), https://www.fda.gov/regulatory‐information/search‐fda‐guidance‐documents/q8r2‐pharmaceutical‐development.

[94] “Measurement Needs for Biofabrication of Tissue Engineered Medical Products Workshop Report ,” can be found under https://phiab.com/publications/measurement‐needs‐for‐biofabrication‐of‐tissue‐engineered‐medical‐products‐workshop‐report/, 2025.

[95] International Vocabulary of Metrology‐Basic and General Concepts and Associated Terms (VIM) , n.d., https://www.nist.gov/document/international‐vocabulary‐metrologypdf.

[96] “NIH Standardized Organoid Modeling (SOM) Center ,” can be found under, https://www.nih.gov/som.

[97] S. Gerke , J. Balamut , and J. K. Wagner , “The FDA's Plan to Phase out Animal Testing,” Trends in Biotechnology 44, no. 8 (2026): 2178–2181, 10.1016/j.tibtech.2025.12.011. PMC12834477 41571550

[98] Booker, C. A. [d‐Nj], FDA Modernization Act 3.0 , 2025, https://www.congress.gov/bill/119th‐congress/senate‐bill/355.

[99] “Commission Accelerates Transition away from Animal Testing in Chemical Safety Assessments ,” can be found under https://ec.europa.eu/commission/presscorner/detail/en/ip_26_1214.

[100] “New Approach Methodologies: NAMs ,” can be found under https://www.pmda.go.jp/english/review‐services/0022.html.

[101] H. Kojima , “History of the Organisation for Economic Co‐Operation and Development (OECD) Test Guidelines for Non‐Animal Test Methods in Japan,” Genes and Environment 47, no. 1 (2025): 3, 10.1186/s41021-024-00323-7. PMC11781050 39881411

[102] 식품의약품안전처, “Ministry of Food and Drug Safety>Information>News&Notice>News&Notice ,” can be found under https://www.mfds.go.kr/eng/brd/m_61/view.do?company_cd=&company_nm=&itm_seq_1=0&itm_seq_2=0&multi_itm_seq=0&page=1&seq=114&srchFr=&srchTo=&srchTp=&srchWord=&utm.

[103] N.‐H. Kang and J. Oh , “The Accomplishments of KoCVAM in the Development and Implementation of Alternative Methods in Korea,” Alternatives to Laboratory Animals 52, no. 3 (2024): 166–176, 10.1177/02611929241244520. 38626463

[104] Policy Interpretation of the NMPA's Opinions on Deepening the Reform of Cosmetics Regulation and Promoting High‐Quality Industry Development, can be found under https://english.nmpa.gov.cn/2025‐12/22/c_1156417.htm.

[105] “The 3Rs – Replacement, Reduction and Refinement ,” can be found under https://www.nhmrc.gov.au/research‐policy/ethics/animal‐ethics/3rs?utm, n.d.

[106] CSIRO , 2023, 10.25919/P5BT-3S39.

[107] A. K. Ressler , G. L. A. Sampaio , S. A. Dugger , et al., “Evidence of Shared Transcriptomic Dysregulation of HNRNPU‐Related Disorder between human Organoids and Embryonic Mice,” Iscience 26, no. 1 (2023): 105797, 10.1016/j.isci.2022.105797. PMC9804147 36594023

[108] S. Kanton , M. J. Boyle , Z. He , et al., “Organoid Single‐Cell Genomic Atlas Uncovers Human‐Specific Features of Brain Development,” Nature 574, no. 7778 (2019): 418–422, 10.1038/s41586-019-1654-9. 31619793

[109] R. M. Agoglia , D. Sun , F. Birey , et al., “Primate Cell Fusion Disentangles Gene Regulatory Divergence in Neurodevelopment,” Nature 592, no. 7854 (2021): 421–427, 10.1038/s41586-021-03343-3. PMC8719633 33731928

[110] Z. He , L. Dony , J. S. Fleck , et al., “An integrated Transcriptomic Cell Atlas of Human Neural Organoids,” Nature 637 (2025): E21. 10.1038/s41586-024-08482-x PMC11711083 39663459

[111] L. Turner , “ISSCR's Guidelines for Stem Cell Research and Clinical Translation: Supporting Development of Safe and Efficacious Stem Cell‐Based Interventions,” Stem Cell Reports 16, no. 6 (2021): 1394–1397, 10.1016/j.stemcr.2021.05.011. PMC8190662 34048693

[112] N. A. Shlobin , M. Sheldon , and S. Lam , “Informed Consent in Neurosurgery: A Systematic Review,” Neurosurgical Focus [Electronic Resource] 49 (2020): E6. 10.3171/2020.8.FOCUS20611 33130611

[113] D. de Jongh , E. K. Massey , and E. M. Bunnik , “Organoids: A Systematic Review of Ethical Issues,” Stem Cell Research & Therapy 13 (2022): 337. 10.1186/s13287-022-02950-9 PMC9308907 35870991

[114] A. L. Bredenoord , H. Clevers , and J. A. Knoblich , “Human Tissues in a Dish: The Research and Ethical Implications of Organoid Technology,” Science 355, no. 6322 (2017): aaf9414, 10.1126/science.aaf9414. 28104841

[115] A. Lavazza , “Potential Ethical Problems With Human Cerebral Organoids: Consciousness and Moral Status of Future Brains in a Dish,” Brain Research 1750 (2021): 147146. 10.1016/j.brainres.2020.147146 33068633

[116] B. J. Kagan , A. C. Kitchen , N. T. Tran , et al., “In Vitro Neurons Learn and Exhibit Sentience When Embodied in a Simulated Game‐World,” Neuron 110, no. 23 (2022): 3952–3969.e8, 10.1016/j.neuron.2022.09.001. PMC9747182 36228614

[117] T. Sawai , H. Sakaguchi , E. Thomas , J. Takahashi , and M. Fujita , “The Ethics of Cerebral Organoid Research: Being Conscious of Consciousness,” Stem Cell Reports 13, no. 3 (2019): 440–447, 10.1016/j.stemcr.2019.08.003. PMC6739740 31509736

[118] M. Kataoka , C. Gyngell , J. Savulescu , and T. Sawai , “The Ethics of Human Brain Organoid Transplantation in Animals,” Neuroethics 16, no. 3 (2023): 27, 10.1007/s12152-023-09532-3. PMC10550858 37809032

[119] A. J. Barnhart and K. Dierickx , “A Tale of Two Chimeras: Applying the Six Principles to Human Brain Organoid Xenotransplantation,” Cambridge Quarterly of Healthcare Ethics 32, no. 4 (2023): 555–571. 10.1017/S0963180123000051 36847198

[120] R. Frodeman , “International Handbook on Responsible Innovation. A Global Resource,” Journal of Responsible Innovation 6, no. 2 (2019): 255–257, 10.1080/23299460.2019.1613612.

